# The Relationship between Aggregation and Toxicity of Polyglutamine-Containing Ataxin-3 in the Intracellular Environment of *Escherichia coli*

**DOI:** 10.1371/journal.pone.0051890

**Published:** 2012-12-14

**Authors:** Gaetano Invernizzi, Francesco A. Aprile, Antonino Natalello, Andrea Ghisleni, Amanda Penco, Annalisa Relini, Silvia M. Doglia, Paolo Tortora, Maria E. Regonesi

**Affiliations:** 1 Department of Biotechnologies and Biosciences, University of Milano-Bicocca, Milan, Italy; 2 Department of Physics, University of Genoa, Genoa, Italy; University of Maryland School of Medicine, United States of America

## Abstract

Several neurodegenerative diseases are triggered by proteins containing a polyglutamine (polyQ) stretch expanded beyond a critical threshold. Among these, ataxin-3 (AT3) is the causative agent of spinocerebellar ataxia type-3. We expressed three authentic AT3 variants in *Escherichia coli*: one normal (AT3-Q24), one expanded (AT3-Q55) and one truncated immediately upstream of the polyQ (AT3-291Δ). Then, based on growth rate reduction, we quantified protein toxicity. We show that AT3-Q55 and -291Δ strongly reduced the growth rate in the early stages (2–4 h), unlike AT3-Q24. This correlated well with the appearance of soluble cytosolic oligomers, but not with the amount of insoluble protein in inclusion bodies (IBs). The impact of AT3-291Δ on cell growth suggests an intrinsic toxicity of the AT3 fragment. Besides the typical Fourier Transform Infrared Spectroscopy (FTIR) signal for intermolecular β-sheets, the expanded form displayed an additional infrared signature, which was assigned to glutamine side-chain hydrogen bonding and associated with SDS-insoluble fibrils. The elongation of the latter was monitored by Atomic Force Microscopy (AFM). This mirrors the well-known *in vitro* two-step aggregation pattern of expanded AT3. We also demonstrated that final aggregates of strains expressing expanded or truncated AT3 play a protective role against toxicity. Furthermore, our findings suggest that the mechanisms of toxicity are evolutionarily conserved.

## Introduction

Protein aggregation into highly stable and structured deposits, the so-called amyloids, is a hallmark of many neurodegenerative diseases [1]. The proteins involved in the onset of the diseases share no sequential or structural similarities. The aggregation process leading to amyloid fibrils, however, involves a common pathway generally triggered by protein misfolding, followed by soluble oligomeric aggregates formation, which in turn are precursors to fibrils [1]. In recent years, plenty of evidence has identified soluble oligomers as the causative agents of cell toxicity [2]–[8], whereas a controversial debate has arisen as to the role of insoluble amyloids as a reservoir rather than sequestering deposit of toxic soluble species [7]–[15]. Interestingly, strategy mechanisms capable of seizing potentially harmful misfolded polypeptides into insoluble deposits seem to be perpetuated throughout the evolution from prokaryotic bacteria to more highly complex organisms [16], [17]. Indeed, in *Escherichia coli*, misfolded proteins are accumulated into inclusion bodies (IBs), insoluble protein macro-aggregates that share several distinctive features with amyloid deposits. Like amyloid fibrils, IBs are structurally organized into intermolecular β-strands, can bind classical amyloid dyes (Thioflavin T, Congo red) and have an assembly, which is driven by selective intermolecular interactions that make the aggregation process highly protein-specific [18], [19]. Furthermore, expression of some amyloidogenic proteins in *E. coli* has resulted in the appearance of amyloid fibrils inside IBs and in the ability of isolated IBs to seed protein fibrillogenesis *in vitro* [20]–[22]. Coupled with its ease of handling, this makes *E. coli* an effective host for fibrillogenesis studies under intracellular conditions that cannot easily be reproduced *in vitro,* such as high molecular crowding, presence of chaperones and proteases, and continuous synthesis of the protein of interest [22]–[25]. In particular, *E. coli* has been successfully employed to monitor *in vivo* the multistep aggregation mechanism of an artificial chimera harboring a polyglutamine (polyQ) tract [25]. Proteins containing stretches of repeated glutamines whose length exceeds a critical threshold undergo amyloid aggregation, which results in neurodegeneration [26], [27]. The aggregation pathway of the most commonly investigated polyQ proteins, huntingtin (Htt) and ataxin-3 (AT3) has been extensively studied *in vitro* and demonstrated to consist of a multistep mechanism involving different domains of the protein [28]–[30]. In particular, regions flanking the polyQ are responsible for the first steps of aggregation, whereas polyQ is involved in a subsequent step leading to the formation of SDS-insoluble fibrillar aggregates. By employing AT3 as a model, we have recently demonstrated that the involvement of the polyQ in the second stage of the aggregation pathway results in the formation of hydrogen bonds among glutamine side chains, which leads to the irreversible formation of SDS-insoluble aggregates [31]. Nevertheless, the multistep aggregation mechanism of an authentic polyQ protein has never been investigated *in vivo,* nor it has been verified whether glutamine-glutamine interaction [31]–[33] is a hallmark of polyQ amyloids in the intracellular environment. Furthermore, the possibility to investigate the aggregation pathway *in vivo* would offer the opportunity to study the intriguing relationship between protein aggregation and toxicity. In this view, *E. coli* has proven to be a convenient host to assess cytotoxicity associated with protein expression. For instance, *E. coli* was found to be sensitive to the protein conformational state, with only the misfolded conformation and soluble aggregates being cytotoxic [34]. In particular, detrimental effects on the cell growth of *E. coli* are reported for the expression of GST harboring an expanded polyQ, as well as for Htt carrying more than 50 glutamine repeats [35], [36]. Here, we characterize for the first time in the intracellular environment of *E. coli* the multistep aggregation mechanism of authentic variants of the polyQ protein AT3. We specifically investigate the relationship between aggregation and cytotoxicity. Using a protein variant carrying an expanded polyQ, we show a correlation between the appearance of soluble species and cytotoxicity, as well as a protective role for insoluble species appearing at the latest stages of the process. We also show that an AT3 variant deprived of polyQ exerts a detrimental effect on cell growth comparable to that of the polyQ expanded variant, which suggests a possible role of the polyQ context in determining cell toxicity.

**Figure 1:**
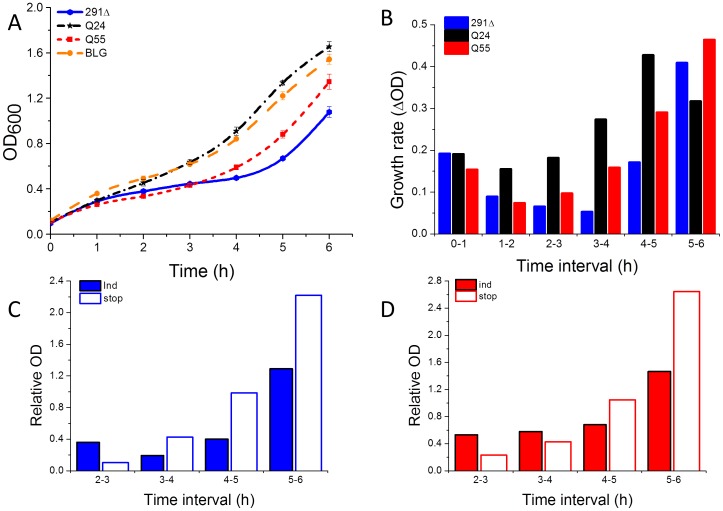
Expression of AT3-291Δ and AT3-Q55 and cytotoxicity. A) Growth curves of *E. coli* strains expressing BLG (yellow dots), AT3-291Δ (blue dots), AT3-Q24 (black stars) and AT3-Q55 (red squares). Cultures were induced with 500 µM IPTG at OD_600_ = 0.1 and growth profiles monitored at 37°C. Error bars represent standard deviations and are derived from at least five independent experiments. B) Changes in optical density per hour (ΔOD) derived from growth profiles of AT3-291Δ (291Δ), AT3-Q24 (Q24) and AT3-Q55 (Q55). C, D) Effect of removal of the inducer on growth rates of AT3-291Δ (C) and AT3-Q55 (D). After 2 h from the induction, cells were resuspended in an inducer-deprived medium (stop, empty bars). As a control, they were further cultured in the presence of the inducer (ind, full bars). ΔODs were normalized with respect to the growth profile of AT3-Q24.

## Materials and Methods

### AT3 Variant Cloning and Expression

AT3 variants were cloned into a pET-21a plasmid (EMD Biosciences) and expressed in *E. coli* Rosetta™ (DE3) pLacI Competent Cells (EMD Bioscience). AT3-Q24 and -Q55 were directly inserted into previously mutagenized NdeI/XhoI restriction sites. AT3-291Δ was obtained by phosphorylated oligonucleotide PCR on Q24 cDNA with the following primers: 291 Δ -Rev 5′ P- TATTTTTCAAAGTAGGCTTCTCGTCTC 3′; 291Δ-For 5′P-TAGCCCGGGTCGACTCGAG 3′. The integrity of the expression hosts was verified by monitoring the growth rate under non-inductive conditions. All *E. coli* strains employed showed identical growth profiles in an LB medium without the inducer Isopropil β-D-1-tiogalattopiranoside (IPTG) (**Fig. S1**).

**Figure 2:**
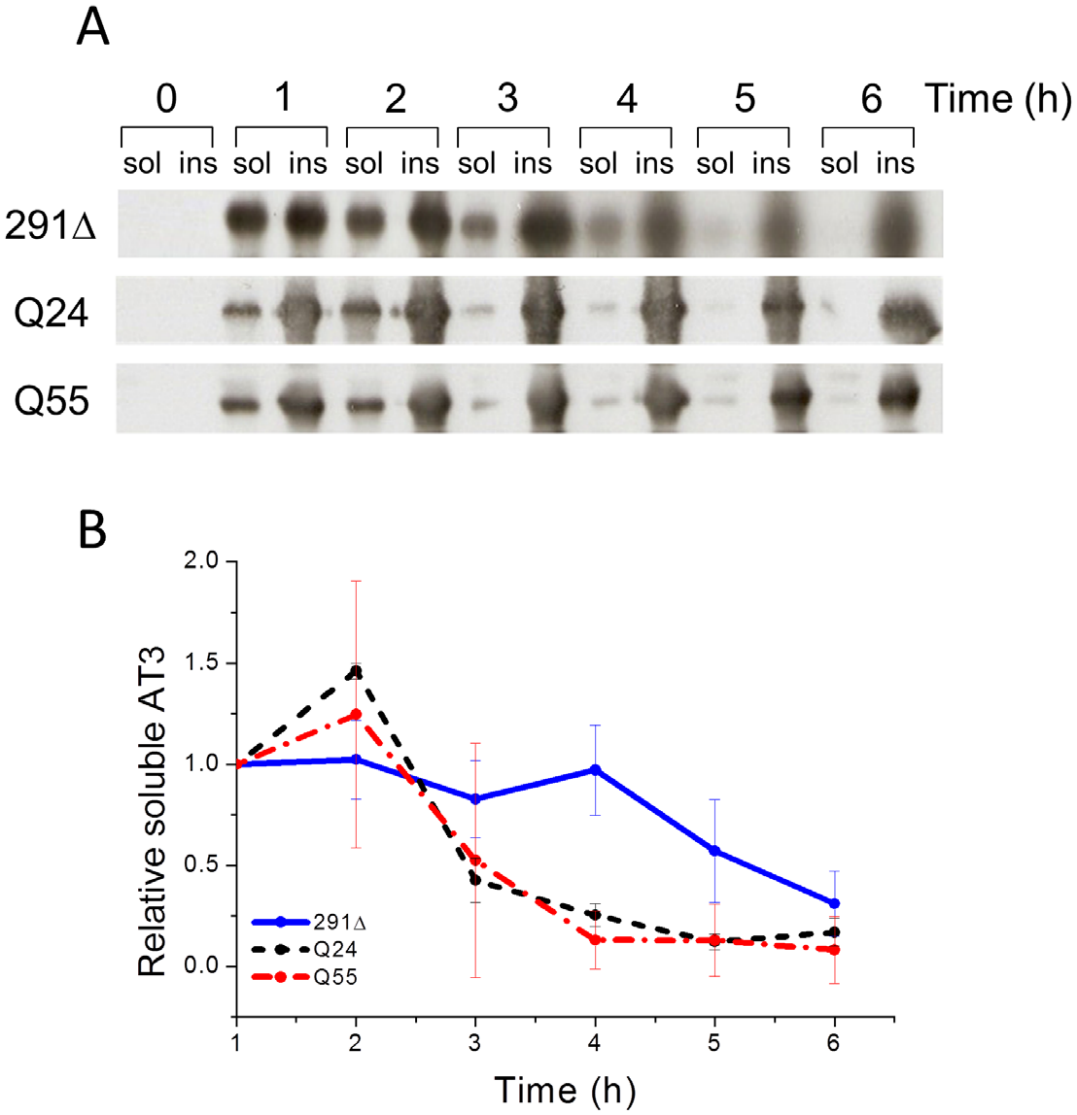
Soluble protein fraction analysis of *E. coli* strains expressing AT3 variants. A) Soluble (sol) and insoluble (ins) fractions of strains expressing AT3 variants were collected at different times after induction, subjected to SDS-PAGE and western blotted using anti-AT3-antibodies. B) Soluble protein fraction was quantified by densitometry of western blots. Signals were normalized at t = 1 h. Error bars represent standard deviations and are derived from at least five independent experiments.

### Growth Profile Analysis

Growth experiments were performed at 37°C, under constant shaking at 150 rpm in an LB standard medium. Cells were induced at OD_600_ = 0.1 with 500 µM IPTG (Qiagen), unless otherwise specified, and growth rates were measured by monitoring OD_600_ at each hour after induction. To study the toxic effect of pre-fibrillar species, 2 h-inducted cells were centrifuged for 10 min at 3,000 g and 37°C, resuspended in the same volume of fresh media deprived of IPTG and analyzed for the growth profile for another 4 h. The protective role of IBs was tested by monitoring the growth profile of 6 h-induced cells upon re-inoculation in a fresh medium at OD_600_ = 0.1.

**Figure 3:**
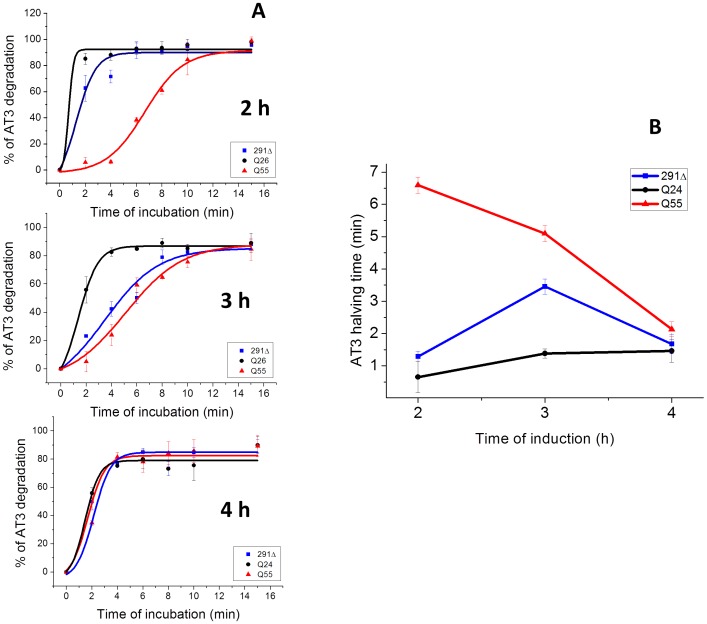
Proteinase K digestion of the AT3 variants in soluble form. A) Soluble AT3 fractions collected at the indicated induction times were incubated for the indicated times at 37°C in the presence of proteinase K (5 µg/ml), subjected to SDS-PAGE and western-blotted using anti-AT3 antibodies. Protein signal was quantified in at least three independent experiments using the ImageJ software. B) AT3 halving times, as determined from the data in Fig. 4A, were calculated as –t50% of the fitting curve y = A0+A/(1+ê((t-t50%)*B)). Error bars represent standard deviations.

### Propidium Iodide Staining of E. coli Cells

To assess viability, cells were subjected to propidium iodide staining. The dye intercalates DNA resulting in enhanced fluorescence emission, but cannot penetrate intact membranes. Cells were centrifuged for 15 min at 6,000 g and 4°C; pellets were then resuspended in 1 volume of 10 mM MgSO_4_ and centrifuged again as above. Resulting pellets were resuspended in the same buffer at an OD_620_ = 0.4 and incubated for 30 min in the dark with 5 µg/ml of propidium iodide, in the absence or presence of 10% isopropanol. The latter treatment resulted in quantitative cell lysis. The samples were centrifuged for 15 min at 6,000 g; pellets were resuspended in the same buffer at an OD_620_ = 0.4 and fluorescence emission determined at 616 nm (excitation: 535 nm) using a Cary Eclipse spectrofluorimeter (Agilent Technologies) in a 3 ml quartz cuvette.

**Figure 4:**
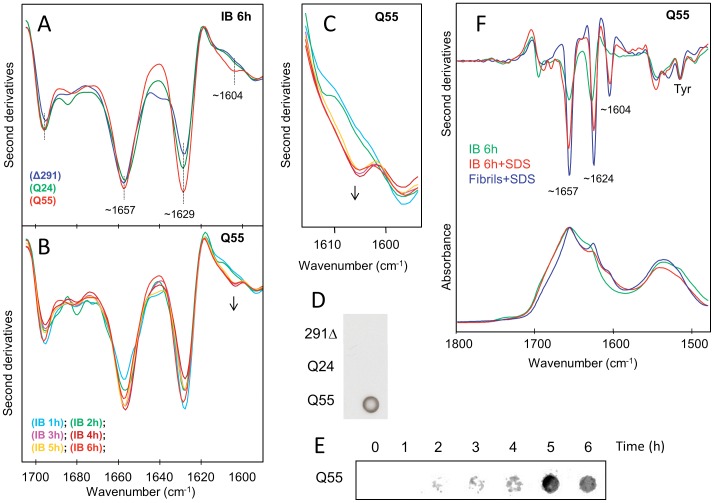
FTIR and filter trap analyses of insoluble protein fractions of *E. coli* strains expressing AT3 variants. A) Second derivative spectra of the insoluble fractions collected at 6 h after induction of the AT3-291Δ, AT3-Q24, and AT3-Q55 expression strains. The peak positions of the main components are indicated. B) Second derivative spectra of the insoluble fractions collected at different time points after induction of the AT3-Q55 expression strain. The arrows point to increasing incubation time of the ∼1,604 cm^−1^ peak, a hallmark of glutamine side-chain hydrogen bonding. C) Second derivative FTIR spectra from (B), reported on an enlarged wavenumber-scale in the spectral region of NH_2_ deformation modes of glutamines. D) Filter trap assay of protein insoluble fractions of the different AT3-291Δ, AT3-Q24 and AT-Q55 after 6 h of induction. E) Filter trap assay of protein insoluble fractions of AT3-Q55 at different times after induction. F) FTIR absorption spectra and their second derivatives of IBs extracted at 6 h from induction are reported before and after SDS-treatment. For comparison, we also give the spectrum of AT3-Q55 SDS-insoluble fibrils, obtained in vitro after incubating the purified protein for 168 h under physiological conditions at 37°C (31). All second derivative spectra are normalized at the ∼1,515 cm^−1^ peak of tyrosine to take possible differences in protein content into account.

### Soluble and Insoluble Protein Fraction Extraction and Inclusion Bodies (IBs) Purification

Cells were harvested for 15 min at 6,000 g and 4°C, frozen at −80°C in 3 volumes of Lysis Buffer (50 mM Tris-HCl, pH 8.0, 100 mM NaCl, 1 mM EDTA) and stored overnight. Then, the cell suspension was thawed; 0.5 mM PMSF and 0.8 mg/ml lysozyme (final concentrations) were added and the resulting mixture incubated at 37°C for 30–45 min, which caused cell lysis. 1% NP-40 was added to the lysate and further incubated at 4°C for 40–60 min. Finally, 25 µg/ml DNase, 10 mM MgSO_4_ were added and the incubation prolonged for 30–40 min. IBs were separated from soluble protein by centrifugation at 10,000 g for 15 min; the pellet was resuspended in 1 ml of Lysis Buffer, 0.5% Triton X-100 and incubated under shaking at 4°C for 15 min. IBs were then collected by centrifuging for 15 min at 10,000 g and 4°C, and washed twice with 1 ml of either milliQ water (for FTIR analysis) or Lysis Buffer, 0.5% Triton X-100 (for all other analyses), in both cases centrifuging at 3,000 g for 15 min. All reagents were from Sigma Aldrich.

**Figure 5:**
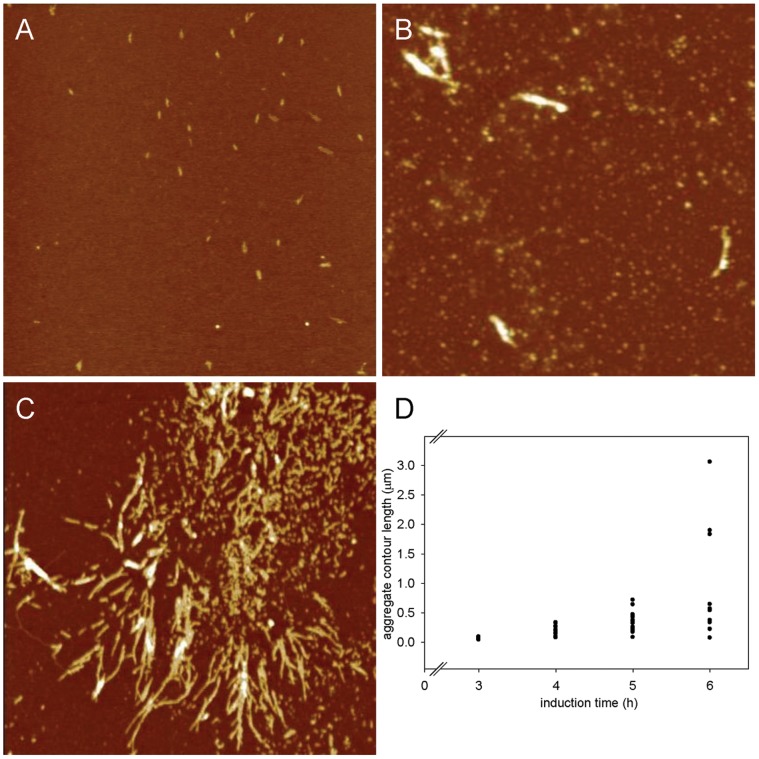
Atomic force microscopy images of AT3-Q55 insoluble protein fraction. A–C) Tapping mode AFM images (height data) of AT3-Q55 insoluble protein fraction after 3 h (A), 5 h (B), 6 h (C) of induction. Scan size 3.0 µm; Z range 5.0 nm (A), 25 nm(B), 15 nm (C). D) Contour length of elongated aggregates (protofibrils and fibrilis) as a function of the induction time.

### SDS-PAGE and Densitometry Analysis of Soluble and Insoluble Protein Fractions

For SDS-PAGE analyses, soluble and insoluble protein fractions from cell culture samples normalized at the same OD were isolated as reported in the IB purification protocol and analyzed in a 14% acrylamide gel. AT3 was revealed by western blotting using an anti-AT3 Z46 rabbit polyclonal antibody [37]. Immunoreactive bands were detected using an ECL Western blotting reagent (GE Healthcare Life Sciences). When required, protein band intensity was quantified using ImageJ software (NIH).

**Figure 6:**
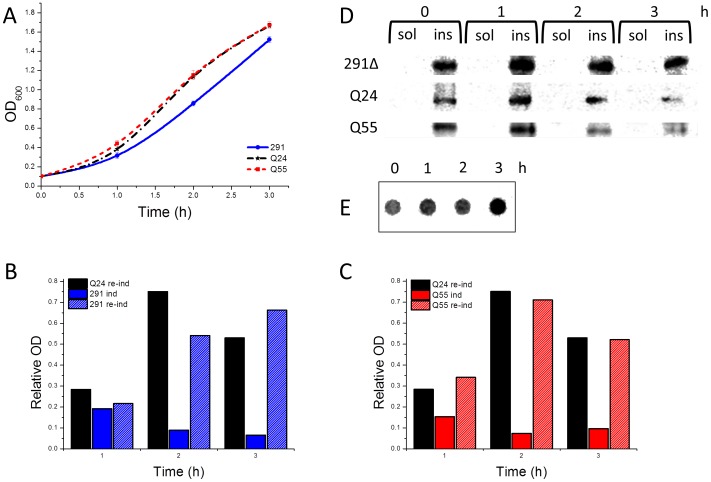
Effect of insoluble aggregates on cytoxicity induced by the expression of AT3 variants. A) Growth curves of *E. coli* strains at 37°C expressing the three AT3 variants. 6 h after the primary induction performed as reported in Fig. 1, cells were harvested and re-inoculated in a fresh medium in the presence of 500 µM IPTG. B, C) Growth rates of AT3-291Δ (B) and AT3-Q55 (C); bars represent growth rates under the primary induction (ind) or during the re-induction (re-ind). For comparison, the growth rate of AT3Q24 under re-induction is also shown. D) Soluble (sol) and insoluble (ins) fractions of strains expressing AT3 variants were collected at different times after re-induction, subjected to SDS-PAGE and western blotted using anti-AT3-antibodies. E) Filter trap assay of protein insoluble fraction of AT3-Q55 at different times after re-induction.

### Pearson Correlation Index

The Pearson correlation index was employed as a measure of the linear correlation between the amount of soluble AT3 and the growth rates of *E. coli* expression strains. It ranges from values of −1 to 1 and was calculated as:
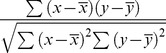
where ‘*x’* and ‘*y*’ are respectively the mean average of *E.coli* growth rates and the soluble protein fraction for a specific AT3 variant.

### Formic Acid Treatment for Inclusion Body Protein Quantification

To solubilize protein from isolated IBs, these were resuspended in 1 ml 100% formic acid and incubated at 37°C for 30 min. Then, protein was determined by absorbance measurement at 280 nm [38].

### Filter Trap Assay and Dot-blot Analysis

Purified IBs from 10 ml growth cultures were resuspended in 500 µl of milliQ water and proteins quantified by formic acid treatment as described above. Samples were centrifuged for 15 min at 10,000 g and 4°C, and pellets resuspended in SDS buffer (20 mM Tris-HCl, pH 6.8, 10 mM DTT, 5% SDS) to a final protein concentration of 0.2 mg/ml. Samples were then boiled for 15 min and 50 µl vacuum filtered in a 48-well dot-blot apparatus through a 0.2 µm pore-sized cellulose acetate membrane. The membrane was washed twice with SDS buffer and probed with an anti-AT3 antibody as reported above.

### Proteinase K Protection Assay

Soluble protein fractions were isolated as reported under the IBs purification paragraph, and 500 µg-samples digested with either 5 or 50 µg proteinase K for 0, 2, 4, 6, 8, 10 and 15 min at 37°C. The enzymatic reaction was stopped by adding 5 mM PMSF and samples were run in 16% SDS-PAGE. AT3 was revealed by anti-AT3 antibody. The extent of AT3 degradation was estimated by densitometry analysis performed by quantifying monomeric band intensity using ImageJ software. Data were fitted using the following sigmoidal equation:
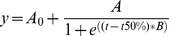
where A_0_ and A are respectively the initial and final values, t50% is the midpoint of the degradation reaction and B is the apparent rate constant of the process.

### Fourier Transform Infrared Spectroscopy

FT-IR measurements on IBs were performed following a previously validated approach [39]. In particular, the extracted insoluble fractions were resuspended in milliQ water and a few microliters of the suspension were deposited onto a BaF_2_ window and dried at room temperature. FT-IR absorption spectra were collected in the transmission mode by coupling the Varian 610-IR infrared microscope – equipped with a nitrogen-cooled Mercury Cadmium Telluride (MCT) detector – to a Varian 670-IR spectrometer (Varian Australia Pty Ltd) under the following conditions: 2 cm^−1^ spectral resolution, 25 kHz scan speed, 512 scan co-additions, and triangular apodization. When necessary, spectra were corrected for possible residual water vapor. The second derivatives of the absorption spectra were obtained after an 11-point binomial smoothing followed by the Savitzky–Golay derivative method (3rd polynomial, 5 smoothing points), using GRAMS/32 software (Galactic Industries).

### Atomic Force Microscopy

For AFM imaging, 10-µl aliquots of the sample obtained from inclusion bodies after washing with SDS were deposited on freshly cleaved mica and then dried under a mild vacuum. AFM measurements were performed in air using a Dimension 3100 scanning probe microscope equipped with a G scanning head (maximum scan size 100 µm) driven by a Nanoscope IIIa controller and a Multimode SPM, equipped with an “E” scanning head (maximum scan size 10 mm) driven by a Nanoscope V controller, (Digital Instruments, Bruker AXS GmbH). Images were acquired in tapping mode in air using single beam uncoated silicon cantilevers (type OMCL-AC160TS, Olympus). The drive frequency was between 290 and 320 kHz and the scan rate was between 0.5 and 1.0 Hz. Aggregate height was measured from the height in cross-section in the topographic AFM images. The length of fibrillar aggregates was measured from the topographic AFM images with a MATLAB (The Mathworks Inc) routine. This routine approximates the fibril profile by using a sequence of segments.

## Results

### The Model System

To provide insight into the relationships between aggregation and toxicity in an intracellular environment, we expressed three authentic AT3 variants, i.e., lacking any tag or fusion partner, in *E. coli*. This is relevant to the goal of our investigation, as it has been demonstrated that the particular protein context of polyQ can greatly influence the aggregation pathway and, consequently, aggregate toxicity [40], [41]. Specifically, we expressed a variant truncated immediately upstream of the polyQ (AT3-291Δ), a wild-type and a pathogenic AT3 variant carrying 24 (AT3-Q24) and 55 (AT3-Q55) consecutive glutamines, respectively. As a control, we used β-lactoglobulin (BLG), a non-amyloidogenic protein, which has been shown to aggregate into inclusion bodies (IBs) when overexpressed in *E. coli* cells [42]. We assessed the expression levels of AT3 variants under the experimental conditions adopted. This was performed by dot-blot analysis of whole cells lysate, which did not show any significant difference (**Fig. S2**).

### AT3-291Δ and AT3-Q55 Expression is Deleterious to Cell Growth

It has been reported that in *E. coli* cell growth and viability are affected by polyQ expression. Furthermore, a correlation between polyQ length and toxicity has been clearly demonstrated [35], [36]. Accordingly, in our model wild-type- and expanded AT3-expression strains displayed substantially different growth profiles (**Fig. 1A and B**), the latter growing much more slowly than the former in the first 3 h after induction. Surprisingly, the strain expressing AT3-291Δ showed a profile very similar to that of the expanded form. Growth profiles of the strains expressing AT3-Q24 and BLG were almost identical to each other, thus showing that the effect on cell growth exerted by wild-type AT3 is merely due to well-described intracellular effects, like energy depletion, which are triggered by protein overexpression [43], [44].

To assess cell viability we performed propidium iodide staining, which is based on the capability of the dye to bind DNA with resulting fluorescence enhancement, but not to permeate intact cell membranes. Cells expressing all AT3 variants failed to appreciably take up the dye. This is consistent with the hypothesis that neither AT3-291Δ nor AT3-Q55 toxicity affects cell viability during the experimental time course (**Fig. S3**). We also attempted to assess cellular toxicity by fluorescence anisotropy assays. This reveals changes in membrane fluidity that are often associated with increased cellular stress [34]. However, none of the strains expressing AT3 displayed significant changes (data not shown).

To confirm that toxic species arising from AT3-Q55 and AT3-291Δ are actually produced in the earliest stages of their intracellular aggregation, we stopped protein induction after 2 h by replacing the growth medium with one deprived of IPTG, and monitored cell growth. Indeed, it has been reported that, following IPTG deprivation, protein expression is strongly inhibited in T7-based inducible strains, like the one employed here [45]. Thus, after IPTG removal we monitored growth rates and normalized them with respect to that of the wild type, to allow for the effect of overexpression-related cellular stress. By comparing them with those of the same strains under induction (**Fig. 1C and D**), it is apparent that after IPTG removal the deleterious effects of the toxic species still persisted for about 4–5 h.

### AT3-291Δ and -Q55 Toxicity Correlates with the Amount of Soluble Protein Fraction

Although the results reported above clearly indicate that the AT3-291Δ and -Q55 variants are toxic to the cell, they alone do not provide any clue as to the identity of the toxic species. In recent years, plenty of evidence has highlighted a critical role for soluble oligomeric amyloid species in triggering cellular toxicity. Actually, detrimental effects of early oligomers have been demonstrated for a number of amyloid proteins including polyQ proteins such as huntingtin and atrophin-1 [6]–[8]. Thus, to settle the issue, we isolated soluble and insoluble protein fractions from our expression strains at different times after induction (see *Experimental Procedures*). In all cases, protein samples from equal cell amounts were loaded. AT3 was revealed by western blot analysis (**Fig. 2A**), and quantified in the soluble fraction by densitometric analysis (**Fig. 2B**). At the first hour after induction soluble AT3 was already detected in all three strains and reached a maximum at 2 h. After that point, soluble AT3-Q55 and AT3-Q24 declined, reaching almost undetectable levels at 4 h. In contrast, in the bacterial strain expressing AT3-291Δ, soluble protein was abundant and essentially constant over the first 3–4 h, significantly decreasing only at later time points (**Fig. 2B**). Remarkably, insoluble protein levels were constantly high in all three strains throughout growth (**Fig. 2A**). Our results highlight two major points: i) AT3-291Δ- and -Q55-expression strains show a remarkable recovery of growth only concomitant to a significant decrease in AT3 soluble fraction. ii) there is no correlation between growth rates and the amounts of insoluble protein detected.

To further substantiate a possible correlation between soluble protein level and toxicity, relative amounts of soluble AT3 were compared with cell growth rates of the respective strains, (**Fig. S4**) at the different sampling times after induction. Then, based on these data, the Pearson Correlation Index was determined. Values of −0.57 and −0.60 were assigned to AT3-291Δ and AT3-Q55, respectively, which highlights a good linear correlation. In contrast, no significant correlation was found for AT3-Q24, as supported by a PcI of −0.20. These results are consistent with the hypothesis that the soluble species responsible for the cytotoxic effects result from protein aggregation of AT3-291Δ and AT3-Q55 in the first 3–4 h after induction.

### AT3-Q55 and -291Δ Soluble Species are More Resistant to Proteinase K Digestion than Wild Type AT3

The results reported above point to the soluble species generated by AT3-291Δ and -Q55 as the culprits for cellular toxicity. This implies that structural differences between toxic and non-toxic oligomers should be detectable.

We therefore subjected to proteinase K digestion the soluble fractions of the three AT3 variants isolated at 2, 3 and 4 h after induction. This made it possible to monitor the time-dependent changes in sensitivity to proteolysis of AT3 soluble species, which was quantified as halving times (t½) of full-length proteins, as detected by western blotting (**Fig. 3A and B and S** **5**). After 2 h, AT3-Q55 soluble species were substantially more resistant to proteinase digestion than those from the two other forms. Subsequently, AT3-Q55 resistance progressively decreased, and after 4 h only marginal differences among the three forms were detectable. AT3-291Δ underwent a significant increase in proteolytic resistance at 3 h from induction compared to 2 h, thus approaching to that of AT3-Q55. AT3-Q24 was instead highly sensitive at any time. As regards the proteolytic fragmentation pattern, qualitative differences among the three forms are clearly apparent (**Fig. S5**) although a detailed interpretation cannot be made given the complexity of such results. It is worthwhile to mention that proteinase K resistance is regarded as a distinctive property of some cytotoxic oligomers [46], [47].

### Structural Characterization of the Insoluble Protein Fraction

To provide insights into the structural features of the insoluble aggregated species, we took advantage of FTIR spectroscopy, a powerful technique that provides information on secondary structure content of species of protein in solution as well as insoluble protein. Also, this technique has been successfully applied to monitor protein aggregation in bacteria [48]. We therefore analyzed insoluble protein fractions from the strains expressing the three AT3 variants. The second derivative spectrum of the insoluble aggregates extracted 6 h after induction is presented in **Fig. 4A**. The spectra of the three AT3 variants displayed well resolved components at 1,657 cm^−1^, assigned to random coils and α-helices, and two bands at 1,696 cm^−1^ and 1,629 cm^−1^ resulting from intermolecular β-sheet structures in protein aggregates. Only in the spectrum of the AT3-Q55 variant, did we observe an additional component around 1,604 cm^−1^, which in a previous study on AT3 fibrillation *in vitro* [31] we had assigned to glutamine side chains involved in a network of hydrogen bonds. Interestingly, we also found that this band is a hallmark of AT3-Q55 SDS-insoluble fibrils. Here, during the time course of the expression of AT3-291Δ and -Q24, no significant spectral variation of the insoluble aggregates was detected by FTIR spectroscopy (**Fig. S6**), indicating that proteins did not undergo major structural rearrangement. Instead, for the AT3-Q55 variant we observed the appearance of the ∼1,604 cm^−1^ component starting from 3 h after induction (**Fig. 4B and 4C**). This shows that structural ordering of the glutamine side chains, involved in a network of hydrogen bonds also occurs *in vivo* at this stage of aggregation. In keeping with our *in vitro* results [31], we expected that the AT3-Q55 aggregates would consist of SDS-insoluble protein. In fact, filter trap assays performed on insoluble protein fractions (**Fig. 4D**) revealed the presence of SDS-resistant species only in the strain expressing AT3-Q55 starting from 3 h after induction (**Fig. 4E**). It is noteworthy that SDS-insoluble material extracted from the IBs displayed an FTIR spectrum almost identical to that collected from the *in vitro* irreversibly aggregated AT3-Q55 (**Fig. 4F**). In particular, both spectra displayed the two components at 1,657 cm^−1^ and 1,604 cm^−1^, respectively due to the C = O and NH vibrations of the glutamine side chains, besides the well resolved band at 1,624 cm^−1^ characteristic of intermolecular β-sheet structures [31].

The SDS-insoluble protein fraction of *E. coli* expressing AT3-Q55 was analyzed by tapping mode AFM at different induction times to get insight into aggregate morphology. Elongated protofibrils with a height of about 1 nm were observed after 3 h (**Fig. 5A**). Longer protofibrils with a height between 2 and 20 nm were present after 5 h (**Fig. 5B**), while after 6 h fibrillar structures with a similar height were also found (**Fig. 5C**). **Fig. 5D** shows the contour length of elongated aggregates (protofibrils and fibrils) measured from the topographic AFM images as a function of the induction time. The aggregate population, which after 3 h of induction had a mean length of 63±9 nm, was gradually enriched with longer structures, some of which reached a few microns at 6 h of induction. Even at the longest induction times the fibrillar structures were found to coexist with globular aggregates. The latter can be identified as oligomeric species.

### AT3-291Δ and -Q55 Soluble Species Toxicity is Strongly Prevented by Insoluble Aggregates

In recent years, increasing evidence has provided support to the idea that cytotoxic species are soluble oligomeric aggregates, and fibrils or amyloid plaques are not likely to be causative agents of pathogenesis. It has also been suggested that they might even exert a protective effect against oligomer toxicity [6]–[10], [14]. Interestingly, *E. coli* IBs are supposed to be a defensive mechanism against potentially harmful misfolded proteins [17], [34]. In keeping with this view, we observed that growth rates of AT3-Q55 and AT3-291Δ-expression cells, after 5–6 h from induction increased up to values comparable to those of non-induced strains in exponential growth (**Fig. 1A** **; Fig. S1**). Actually, in this lapse of time the aggregated species detected intracellularly were almost exclusively insoluble (**Fig. 2A**).

To assess whether insoluble aggregates could also exert a protective effect against soluble species toxicity, strains expressing AT3 variants grown for 6 h under inducing conditions were re-inoculated at the initial optical density (OD_600_ = 0.1) into a fresh new medium, still in the presence of IPTG. After re-induction, growth rates of AT3-291Δ- and AT3-Q55-expression strains were substantially higher than those observed after the primary induction (**Fig. 6A**; for comparison see **Fig. 1A**) and comparable to those of re-induced AT3-Q24 (**Fig. 6B and C**), which points to a protective role of the insoluble aggregates against the soluble toxic species. It should also be noted that growth rates of re-induced AT3-Q24 were higher than those observed during the primary induction, which could be accounted for by the massive recruitment into IBs of soluble misfolded protein. This has already been reported in the case of another nontoxic protein expressed in the prokaryotic host [34]. Cell fractionation of re-induced AT3-291Δ and -Q55 showed the massive presence of aggregated species at all the time points analyzed, whereas soluble species were hardly detectable (**Fig. 6D**). SDS-insoluble species were present over the entire re-induction time (3 h) in the strain expressing AT3-Q55 (**Fig. 6E**). Furthermore, it is interesting to note that AFM images of an insoluble AT3-Q55 fraction after 6 h of induction revealed the presence not only of fibrillar aggregates but also of oligomeric species that were trapped inside the IBs (**Fig. 5C**).

## Discussion

Plenty of evidence collected in recent years has shown that several neurodegenerative diseases are associated with the accumulation of amyloid aggregates [1]. Each disease normally results from the enhanced propensity of one specific protein to aggregate, although the proteins involved are unrelated to each other [1]. Initially, this led to the conclusion that such macromolecular assemblies are the causative agents of the pathologies [49]. However, compelling evidence achieved more recently attributes cell toxicity to the oligomeric species arising in the early stages of fibrillogenesis [2]–[8]. Thus, to provide insight into the mechanisms underlying toxicity, a thorough understanding of aggregation pathways and structural features of the intermediates is required. Actually, *in vitro* investigations on fibrillogenesis of different amyloid proteins such as α-synuclein, β-amyloid and Htt, have helped identify the species formed during the process [15], [29] **.** However, the intracellular environment might greatly influence the aggregation mechanisms, as a result of: i) high protein concentration and a macromolecular crowding effect, ii) presence of chaperones and proteases and iii) constant supply of polypeptides by the cellular translational complex. This implies that the aggregation intermediates isolated *in vitro* might not be representative of those generated intracellularly. Based on these considerations, studies *in vivo* are not only required to identify the cellular targets of the toxic species, but also to establish whether and how the aggregation pathway is affected by the intracellular environment.

*E. coli* has been long exploited as a useful cellular model to study aggregation of amyloid-related proteins in a physiological background and also to screen anti-amyloidogenic compounds [18]–[20], [22], [23], [25], [50]–[53]. In particular, it was also employed to study the aggregation pattern of a polyQ chimera, which highlighted a multidomain misfolding pathway resembling that followed *in vitro* by the authentic polyQ proteins AT3 and Htt [25]. However, investigations on artificial constructs are biased by the effect of polyQ-flanking sequences, which in fact can deeply influence the aggregation pathway and, consequently, the toxicity exerted by the protein [40], [41]. This is why authentic polyQ proteins should be assayed for such studies, although the use of artificial constructs may be justified by the requirement of a physically detectable tag to monitor aggregation.

Based on these premises, we assessed the toxic effects of authentic AT3-Q24 and AT3-Q55, i.e. a normal and expanded polyQ protein, respectively, by expressing them in the prokaryotic host. To the best of our knowledge, this is the first report and toxicity analysis of authentic full-length polyQ proteins expressed in an intracellular milieu. To detect possible polyQ-unrelated effects, we also expressed AT3-291Δ a variant truncated immediately upstream of the polyQ itself. The structural features of the intermediates were characterized in cell lysates by several biochemical and biophysical techniques. In particular, FTIR made it possible to detect the changes in secondary structural content and, most important, the increase in protein-protein interactions. Cellular toxicity was quantified on the basis of the decrease in growth rates and correlated with the appearance of different aggregated species. Also, our results strongly suggest that the effect of the toxic oligomers mostly resulted in a bacteriostatic rather than a bactericidal effect, as substantiated by cell inability to take up propidium iodide at any time (**Fig. S3**).

When expressed in *E. coli*, expanded AT3 aggregated essentially in the same way as *in vitro* [31], namely undergoing a two-step fibrillogenesis process, the first giving rise to SDS-soluble oligomers, the second to SDS-insoluble fibrils found in the IBs. In FTIR, the latter displayed the typical pattern of side-chain glutamine hydrogen bonding, a hallmark of irreversible polyQ aggregation [31]. AFM highlighted the fibrillar morphology of these aggregates, which elongate during the experimental time frame, providing a direct representation of the polyQ fibrils formed. These findings are the first evidence of a multidomain misfolding of an authentic amyloid protein in an intracellular environment [54]. As expected, AT3-Q24 and AT3-291Δ underwent the first step of aggregation, but did not generate any SDS-insoluble aggregate, as supported by filter trap assays, although aggregation products were found in the IBs as well.

Time courses of soluble protein showed almost identical profiles for AT3-Q24 and AT3-Q55, with a maximum around 2 h, followed by a relatively fast decline (**Fig. 2**). This result conforms to the “induced misfit” model [55], [56] that assigns similar intrinsic aggregation rates to normal and expanded polyQ proteins, but assumes that the former is prevented from aggregating via interactions with its physiological partners, which of course cannot take place in the prokaryotic cell. In contrast, the level of soluble AT3-291Δ declined much more slowly than that of the two other forms until 5–6 h after induction. Thus, it showed comparatively higher levels at the latest times (3–5 h). Interestingly, this matches well with the *in vitro* observation that AT3-291Δ [57] undergoes slower aggregation kinetics with respect to those of AT3-Q24 and AT3-Q55 [31].

When quantifying the toxic effects of the three proteins, we demonstrated that, as expected, the expanded AT3 dramatically reduced growth rate while the normal AT3 did not. Strikingly, we observed that the truncated variant was also toxic, which suggests a direct involvement of the polyQ-harboring context in protein toxicity. Recently, a study on an animal model demonstrated that mice, both homozygous and heterozygous for an AT3 variant truncated at Gly259, developed severe motor coordination dysfunction and altered behavior, followed by premature death [58]. The pathology was associated with extranuclear protein inclusions, massive neuronal cell death and altered ERAD response. Thus, despite the obvious differences between prokaryotic and eukaryotic cellular environments, truncated AT3 exhibits toxic effects in both systems, which suggests a conserved gain-of-function mechanism of toxicity triggered by proteolysis.

Growth rates of cells expressing AT3-291Δ or AT3-Q55, but not AT3-Q24, displayed a significant inverse correlation with the levels of early soluble oligomeric species. Remarkably, in the case of cells expressing AT3-Q55, not only was the cytotoxic effect apparent prior to the formation of SDS-insoluble, fibrillar aggregates, but also their later accumulation did not affect cell growth. These findings are in line with the hypothesis that the oligomeric species formed in the first step of the aggregation process are the culprits of cellular toxicity but the final aggregates are not.

As mentioned above, the wild-type AT3-Q24 variant should be regarded rather as nontoxic, since in any case, its marginal impact on growth rate could be fully accounted for by its massive overexpression. As a consequence, the soluble species arising from its aggregation must be devoid of any detrimental effect. The most straightforward explanation might be that the aggregation pathway of AT3-Q24 leads to the formation of soluble oligomeric intermediates that are structurally different from those generated by AT3-Q55 and AT3-291Δ. To shed light on this critical issue, we characterized proteinase K resistance of the soluble aggregation products generated by the AT3 variants, as this property has been associated with fibrillar-like oligomeric structures and toxicity [59]. We observed that AT3-Q55 and -291Δ soluble aggregates are more resistant than those resulting from AT3-Q24, except in the last time of induction (4 h). This loss in proteinase resistance can be plausibly justified by the recruitment of a part of soluble species into the insoluble IBs. Interestingly, two recent paper show that α-synuclein and prion cytotoxic oligomers also possess proteinase K resistance and fibrillar-like oligomeric structure [46], [47]. However, at the present stage of our investigations we have no clear explanation to account for the major differences in toxicity we detected among the AT3 variants. This will therefore be a subject of our further investigations. In any case, these findings provide an important clue to the issue of the lack of toxicity of wild-type AT3, despite its capability to give rise to soluble oligomers [30], [59]. Based on our results, this should be related to their intrinsic structural properties rather than to different intracellular abundances of the oligomers generated by the normal form and the expanded form. No less interestingly, we observed that the final aggregates could largely prevent cellular toxicity resulting from soluble oligomer accumulation, irrespective of whether they were SDS-insoluble (AT3-Q55) or not (AT3-291Δ). This is clearly supported by an experiment, whereby the two variants were re-induced after the primary induction, a condition that leads to protein new synthesis in the presence of insoluble aggregates (**Fig. 6**). This result adds direct evidence to the controversial debate on the protective role of intracellular inclusions in polyQ diseases [7]–[15]. In any case, our report makes it possible to rule out that the culprits of cell toxicity in the early growth stages are IBs, which is, however, the case of Aβ42, as recently shown by Ventura and coworkers [20]. They demonstrated that IBs formed in *E. coli* by this peptide are toxic to mammalian cells in culture, due to a massive presence of oligomeric structures as opposed to prefibrillar species rich in intermolecular β-sheet interactions. However, IBs generated by AT3-291Δ and AT3-Q55 (**Figs. 4B and S** **6**) display similar secondary structure content and intermolecular β-sheet interactions throughout the experimental time-course, which suggests that changes in cell growth rates are not caused by conformational changes occurring in IBs.

In conclusion, we showed that the expanded and truncated but not the normal AT3 exert cytotoxic effects in *E. coli*, which is in line with plenty of reports on polyQ protein toxicity in higher organisms, including humans [40], [58], [60], [61]. In our system, we were also able to reproduce the intracellular two-step aggregation pathway of the expanded AT3 and detect the toxic effect of the oligomeric species. These findings suggest that the mechanisms of toxicity are essentially conserved throughout the evolutionary tree. In addition they show that *E. coli* is a valuable model organism for studying amyloid aggregation in the intracellular environment using authentic polyQ proteins thanks in part to its ease of handling.

## Supporting Information

Figure S1 **Comparison of AT3 expression strains under non-inducing conditions.** Growth curves of AT3-291Δ (red), AT3-Q24 (green), AT3-Q55 (blue) and BLG (yellow) expression strains at 37°C.
(TIFF)

Figure S2 **Dot-blot analysis of AT3 expression.** A) Whole protein extracts of *E. coli* strains expressing the AT3 variants at 1 and 3 h after induction were boiled with PBS, 5% SDS buffer and dot-blotted using the anti-AT3 antibody. B) Signal quantification was carried out with Image Studio Analysis (Li-cor) using Bg lane as a background (signal 1.78 E^−4^) (panel B). Error bars represent standard deviations and are derived from three independent experiments.
(TIF)

Figure S3 **Propidium iodide staining of AT3-expression strains.** Fluorescence emission of propidium iodide in strains expressing the three AT3 variants and in cells treated with 10% isopropanol as a positive control (C+). Error bars represent standard deviations and are derived from at least five independent experiments.
(TIF)

Figure S4 **Comparison between soluble amounts and growth rates of AT3 variants.** Growth rates of AT3 expression strains from figure 1B (lines) were plotted with relative amounts of the soluble protein fraction produced (columns). Error bars represent standard deviations and are derived from at least five independent experiments.
(TIFF)

Figure S5 **Proteinase K digestion of AT3 variants.** Soluble AT3 fractions, collected at the indicated induction times, were incubated for different times at 37°C in the presence of proteinase K (5 µg/ml), subjected to SDS-PAGE and western-blotted using anti-AT3 antibodies.
(TIF)

Figure S6 **FTIR characterization of initial and mature aggregates of AT3-291Δ and AT3-Q24.** Second derivative FTIR spectra of the insoluble fractions collected at 1 and 6 h after induction of the AT3-Δ291 (A) and AT3-Q24 (B) expression strains. Spectra are normalized at the ∼1515 cm^−1^ peak of tyrosine.
(TIFF)
