# Supplementary figures and images for: The Relationship between Aggregation and Toxicity of Polyglutamine-Containing Ataxin-3 in the Intracellular Environment of Escherichia coli
Source: PLoS One. 2012 Dec 14;7(12):e51890. doi: 10.1371/journal.pone.0051890 (PMC3522584; doi:10.1371/journal.pone.0051890)

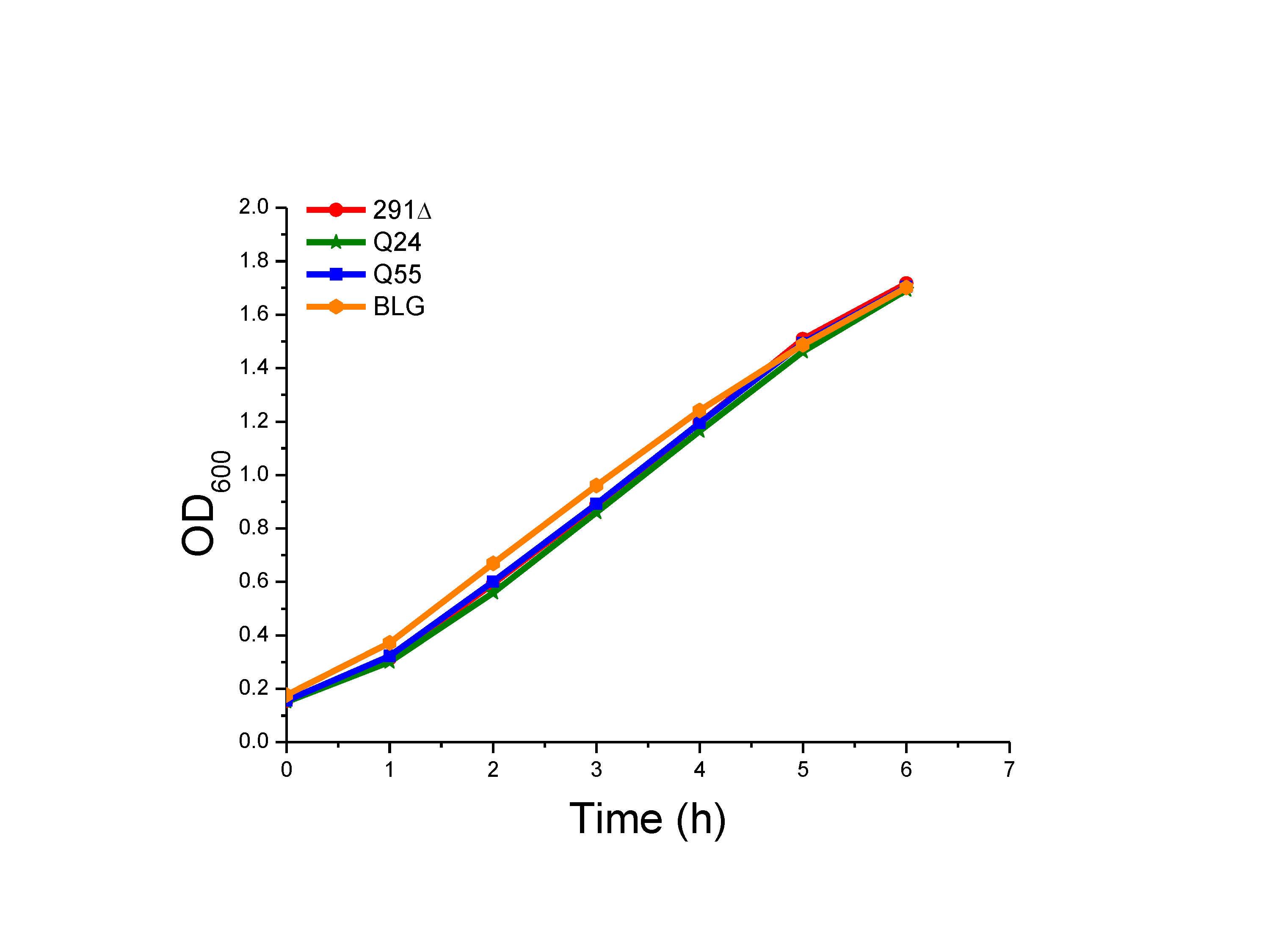

Supplement: Figure S1 — Comparison of AT3 expression strains under non-inducing conditions. Growth curves of AT3-291Δ (red), AT3-Q24 (green), AT3-Q55 (blue) and BLG (yellow) expression strains at 37°C. (TIFF) [file pone.0051890.s001.tiff]

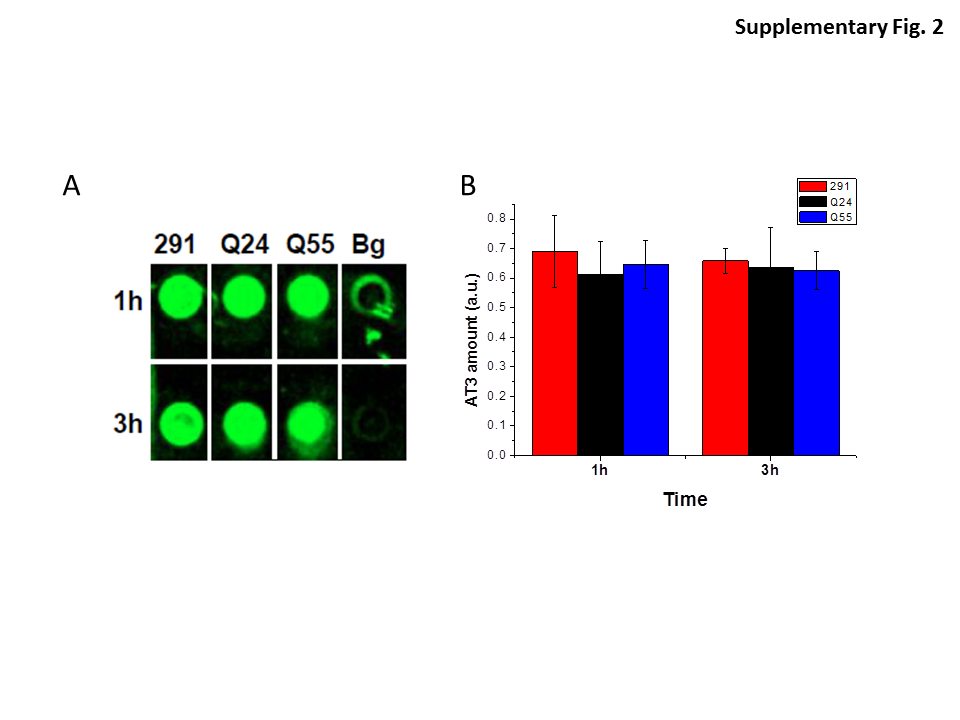

Supplement: Figure S2 — Dot-blot analysis of AT3 expression. A) Whole protein extracts of E. coli strains expressing the AT3 variants at 1 and 3 h after induction were boiled with PBS, 5% SDS buffer and dot-blotted using the anti-AT3 antibody. B) Signal quantification was carried out with Image Studio Analysis (Li-cor) using Bg lane as a background (signal 1.78 E−4) (panel B). Error bars represent standard deviations and are derived from three independent experiments. (TIF) [file pone.0051890.s002.tif]

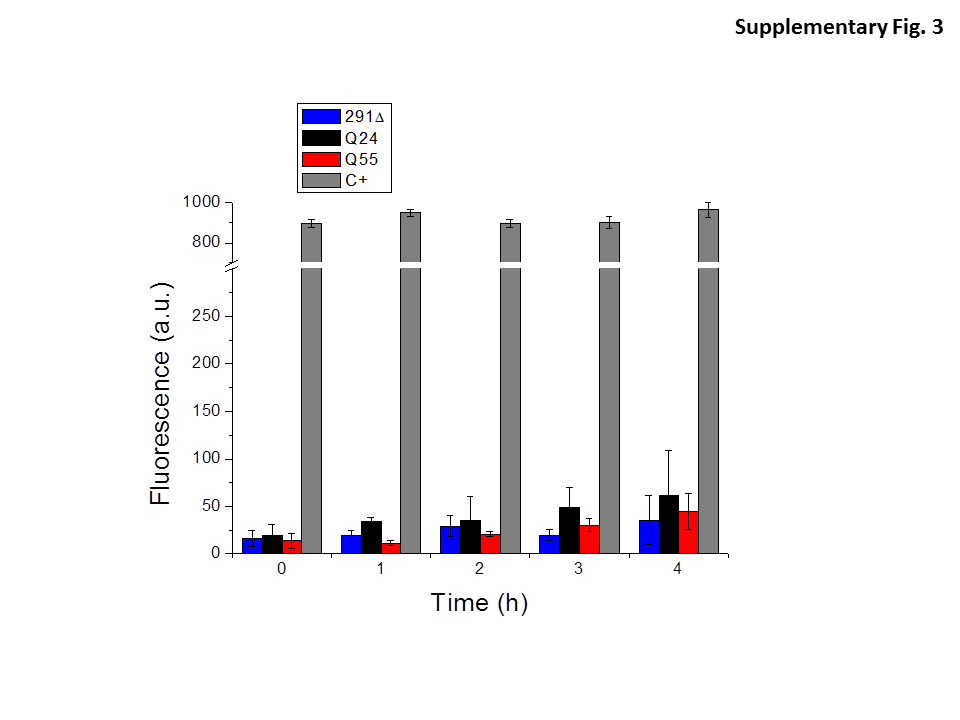

Supplement: Figure S3 — Propidium iodide staining of AT3-expression strains. Fluorescence emission of propidium iodide in strains expressing the three AT3 variants and in cells treated with 10% isopropanol as a positive control (C+). Error bars represent standard deviations and are derived from at least five independent experiments. (TIF) [file pone.0051890.s003.tif]

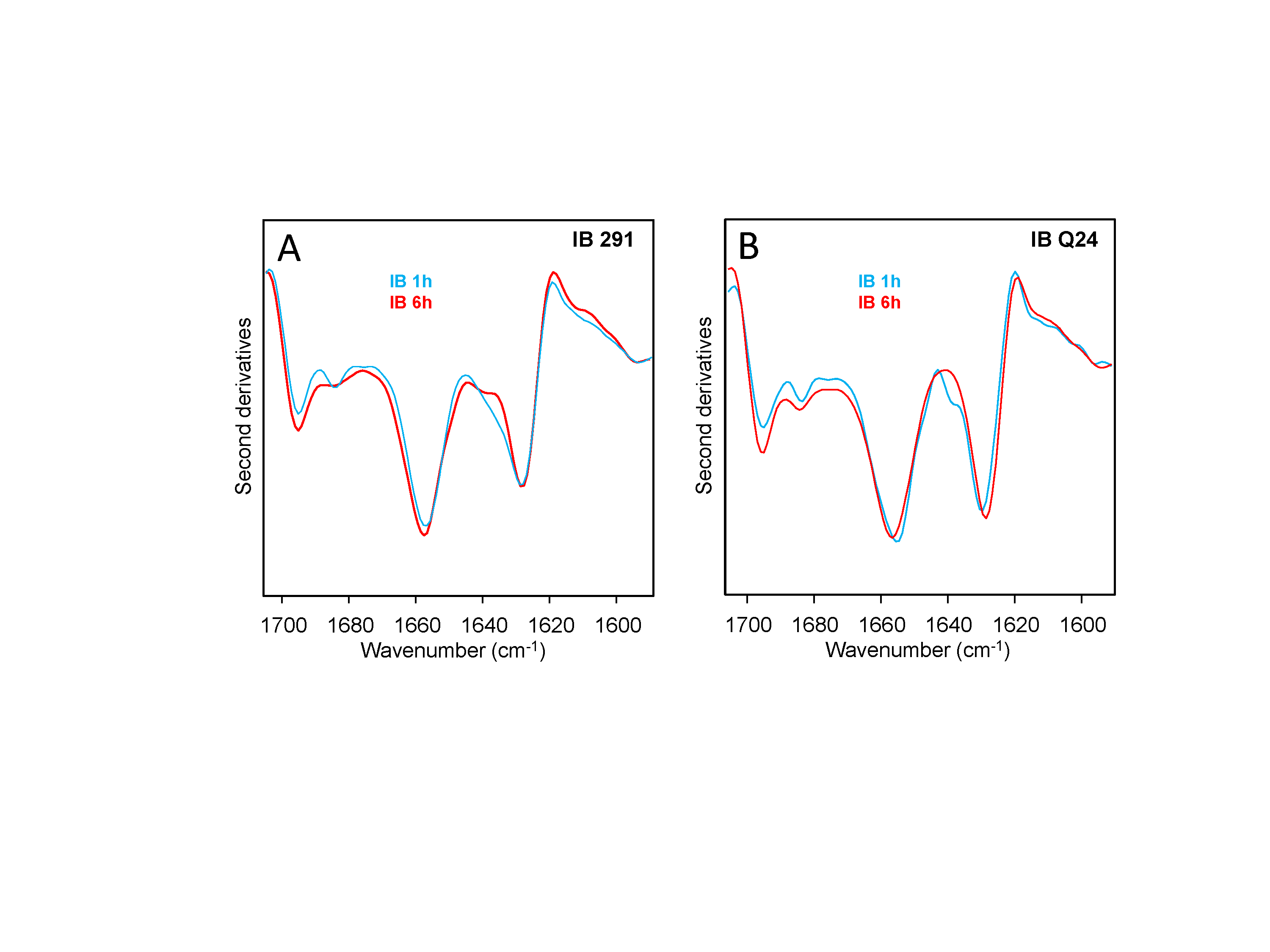

Supplement: Figure S6 — FTIR characterization of initial and mature aggregates of AT3-291Δ and AT3-Q24. Second derivative FTIR spectra of the insoluble fractions collected at 1 and 6 h after induction of the AT3-Δ291 (A) and AT3-Q24 (B) expression strains. Spectra are normalized at the ∼1515 cm−1 peak of tyrosine. (TIFF) [file pone.0051890.s006.tiff]
